# Genetic analysis of a 66-kDa protein-encoding gene of *Angiostrongylus cantonensis* and *Angiostrongylus malaysiensis*

**DOI:** 10.1017/S0031182022001573

**Published:** 2023-01

**Authors:** Abdulhakam Dumidae, Chanakan Subkrasae, Jiranun Ardpairin, Aunchalee Thanwisai, Apichat Vitta

**Affiliations:** 1 Department of Microbiology and Parasitology, Faculty of Medical Science, Naresuan University, Phitsanulok 65000, Thailand; 2 Centre of Excellence in Medical Biotechnology, Faculty of Medical Science, Naresuan University, Phitsanulok 65000, Thailand; 3 Center of Excellence for Biodiversity, Faculty of Sciences, Naresuan University, Phitsanulok 65000, Thailand

**Keywords:** 66-kDa protein, *Angiostrongylus*, genetic diversity, haplotype, phylogeny

## Abstract

The rat lungworm *Angiostrongylus cantonensis* is globally known to be the cause of oeosinophilic meningitis in humans. Another congener, *Angiostrongylus malaysiensis*, is closely related to *A. cantonensis* and has been described as a potential human pathogenic parasite. These 2 worms are similar in terms of life cycle, host range and morphological and genetic information. However, there are limited studies about their genetic diversity based on the 66-kDa protein-encoding gene. The objective of this study was to explore the 66-kDa protein sequence variation of *A. cantonensis* and *A. malaysiensis* collected from Thailand. Two adult and 53 third-stage larval specimens of *Angiostrongylus* from 4 geographic locations in Thailand were molecularly identified using the 66-kDa protein gene. The phylogenetic trees (Bayesian inference tree and maximum-likelihood tree) showed that *Angiostrongylus* formed a monophyletic clade with a clear separation between *A. cantonensis* and *A. malaysiensis*. The genetic distance between *A. cantonensis* and *A. malaysiensis* varies from 0.82 to 2.86%, with a total of 16 variable sites. The analysis of genetic diversity revealed 1 and 5 new haplotypes of *A. cantonensis* and *A. malaysiensis*, respectively, and showed genetic differences between the populations of *A. cantonensis* and *A. malaysiensis*. The haplotype networks of *A. cantonensis* and *A. malaysiensis* populations in Thailand are similar to those of populations in some countries, indicating the range expansion of genomic origin between populations in different areas. In conclusion, the 66-kDa protein gene was a good genetic marker for studying genetic diversity and discriminating between *A. cantonensis* and *A. malaysiensis*.

## Graphical abstract

**Figure:**
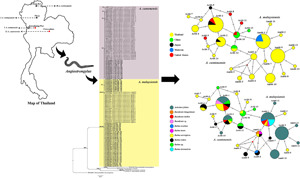

## Introduction

*Angiostrongylus* Kamensky, 1905 or the lungworm is a parasitic nematode in the superfamily Metastrongyloidea (Spratt, 2015). To date, over 20 species of this genus have been reported around the world, and *Angiostrongylus cantonensis* and *Angiostrongylus costaricensis* have been reported to be causative agents of neurological and abdominal angiostrongyliasis in humans, respectively (Spratt, 2015; Barratt *et al*., 2016). Another species found in Asian countries, *Angiostrongylus malaysiensis*, is also a potential human pathogenic parasite (Ansdell and Wattanagoon, 2018). In Thailand, *A. cantonensis* and *A. malaysiensis* are the most common species (Watthanakulpanich *et al*., 2021). *Angiostrongylus cantonensis* is a well-known pathogen that causes oeosinophilic meningitis associated with angiostrongyliasis in humans in Thailand, whereas *A. malaysiensis* is increasingly reported in many provinces in the country (Eamsobhana, 2013; Watthanakulpanich *et al*., 2021).

*Angiostrongylus cantonensis* and *A. malaysiensis* are similar in terms of life cycle, host range, host habitat and morphological and genetic information (Bhaibulaya, 1979; Eamsobhana *et al*., 2015; Chan *et al*., 2020). These 2 species of *Angiostrongylus* can infect the same species of definitive and intermediate hosts (Bhaibulaya and Techasoponmani, 1972). In addition, mixed infections of *A. cantonensis* and *A. malaysiensis* in snail intermediate hosts and rodent definitive hosts have been widely reported (Eamsobhana *et al*., 2016; Watthanakulpanich *et al*., 2021). This may lead to difficulty in discriminating between *A. cantonensis* and *A. malaysiensis*. Adults of *A. cantonensis* and *A. malaysiensis* can be morphologically differentiated by the minute protrusion at the posterior end of females and the bursal rays of males (Bhaibulaya, 1979; Thiengo *et al*., 2010). Nevertheless, the morphological variance between the 2 species can confound identification. Moreover, differences between the morphological characteristics of the larval stages, especially the infective stage, have not yet been clarified. Therefore, the identification of *Angiostrongylus* species based on morphological characters is difficult due to vague and similar descriptions of size and body shapes among species (Robles *et al*., 2008; Monte *et al*., 2014).

Recently, molecular analysis has been used to differentiate various *Angiostrongylus* species (Chan *et al*., 2020; Anettová *et al*., 2022). Molecular and phylogenetic studies for discriminating closely related *Angiostrongylus* spp. have used mitochondrial and nuclear genes as genetic markers; the mitochondrial genes include the cytochrome c oxidase subunit I (COI), cytochrome b (cytb), 12S rRNA and 16S rRNA genes (Rodpai *et al*., 2016; Dumidae *et al*., 2019; Chan *et al*., 2020), and the nuclear genes include the 66-kDa protein gene, small subunit ribosomal RNA (18S rRNA) gene and internal transcribed spacer 2 (ITS2) region (Eamsobhana *et al*., 2010, 2019; Rodpai *et al*., 2016; Dumidae *et al*., 2019). Furthermore, the whole mitochondrial genome has been employed for phylogenetic analysis and species distinction (Valentyne *et al*., 2020).

Molecular phylogeography analysis of *A. cantonensis* and *A. malaysiensis* may provide insight into specific genetic variation and population formation (Avise, 2000). The phylogeography based on COI sequences of *A. cantonensis* from Thailand, Taiwan, China and Japan revealed that the geographical distribution of *A. cantonensis* probably reflects multiple independent origins that were likely to have been influenced by human activities (Tokiwa *et al*., 2012). Likewise, Monte *et al*. (2012) analysed the phylogenetic relationship of COI sequences for *A. cantonensis* and revealed that some haplotypes from Brazil clustered with isolates from Asia, while the rest formed distinctly divergent clades, indicating multiple origins of *A. cantonensis* in Brazil. In addition, the phylogeny based on the 66-kDa protein gene revealed distinct clades among *A. costaricensis*, *A. cantonensis* and *A. malaysiensis*. However, no clear separation of the conspecific taxa between *A. cantonensis* and *A. malaysiensis* from different geographical regions was reported. Greater sample sizes of the conspecific taxa from each locality may provide a conclusive inference of distinct phylogeographic patterns (Eamsobhana *et al*., 2019). Moreover, there have been no reports on the molecular identification of third-stage larvae of *A. cantonensis* and *A. malaysiensis* using the 66-kDa protein gene. Therefore, we further analysed the genetic diversity of *A. cantonensis* and *A. malaysiensis* in Thailand using 66-kDa protein gene sequences. The phylogeny and haplotype network of a 66-kDa protein gene in *A. cantonensis* and *A. malaysiensis* were also analysed to determine the relationship between hosts and parasites.

## Materials and methods

### *Angiostrongylus* worms

**Fig. 1.**
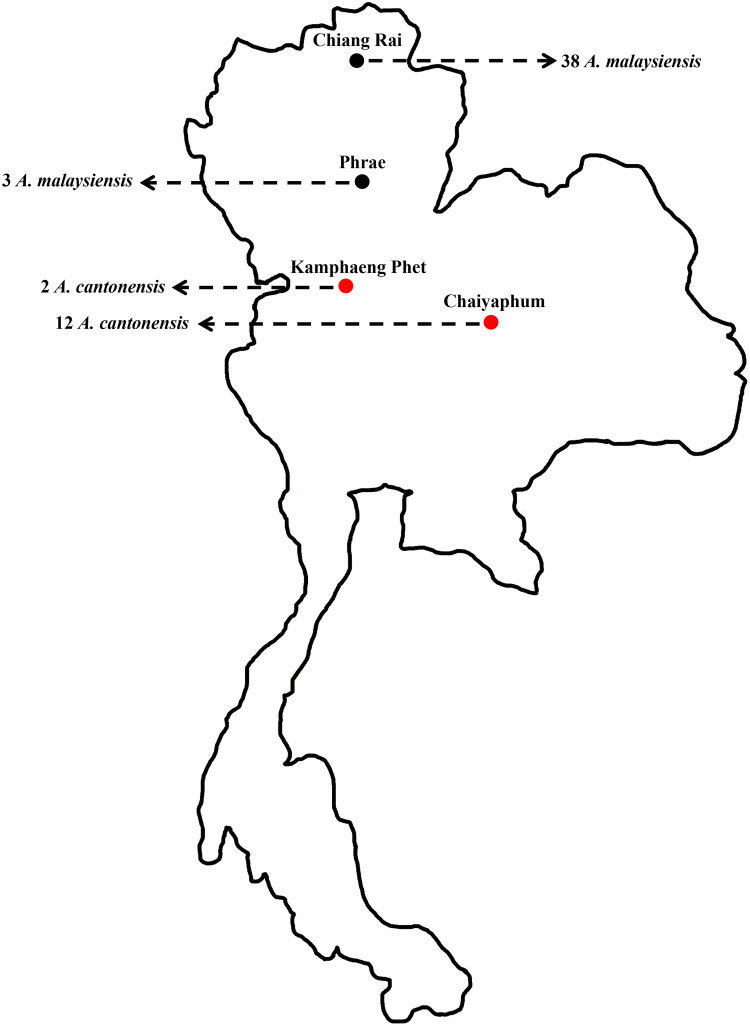
Map of Thailand showing the number of samples of representative *Angiostrongylus* spp. The red and black circles indicate *Angiostrongylus cantonensis* and *Angiostrongylus malaysi*ensis, respectively.

The total 55 *Angiostrongylus* samples consisted of 14 specimens of *A. cantonensis* and 41 specimens of *A. malaysiensis*. Within the 14 specimens of *A. cantonensis*, 2 female worms were collected from a definitive rodent host (*Bandicota* sp.; *n* = 1) in Kamphaeng Phet province, central Thailand, and 12 third-stage larvae (L3) were previously collected from an intermediate land snail host (*Achatina fulica*; *n* = 30) from Chaiyaphum province, northeastern Thailand (Dumidae *et al*., 2019). Of the 41 *A. malaysiensis* samples, 38 L3 specimens were collected from 21 *A. fulica* in Chiang Rai province, and the remaining 3 L3 specimens were collected from 1 *A. fulica* in Phrae province in northern Thailand (Dumidae *et al*., 2019), as shown in Fig. 1. Adult worms were fixed in absolute alcohol and stored at −20°C until DNA extraction. The genomic DNA samples of *Angiostrongylus* larvae were stored at −20°C.

### DNA extraction

Before performing the DNA extraction, adult worms were dried by placing on filter paper for 15 min at room temperature. Individual worms were excised into small pieces, which were then placed in 1.5 mL microcentrifuge tubes, crushed and digested in lysis buffer. Genomic DNA extraction was performed using the NucleoSpin^®^ Tissue kit (Macherey-Nagel, Duren, Germany) according to the manufacturer's protocol. Genomic DNA samples of *Angiostrongylus* larvae that had been collected previously (Dumidae *et al*., 2019) from *A. fulica* were used as samples in this study. The genomic DNA was checked by running it on a 0.8% agarose gel in 1× TBE buffer at 100 V. The gel was stained with ethidium bromide, destained with distilled water and photographed under ultraviolet light. The DNA solution was stored at −20°C prior to further processing.

### Polymerase chain reaction (PCR) and sequencing

The DNA fragment (300 bp) of the 66-kDa protein gene was amplified using primers AC1 5′-CTCGGCTTAATCTTTGCGAC-3′ and AC2 5′-AACGAGCGGCAGTAGAAAAA-3′ (Eamsobhana *et al*., 2019). The PCR components (30 *μ*L final reaction volume) contained 15 *μ*L of EconoTaq^®^ PLUS 2× Master mix (1×; Lucigen Corporation, Middleton, WI, USA), 1.5 *μ*L of each primer at 5 *μ*m (0.25 *μ*m), 9 *μ*L of distilled water and 3 *μ*L of the DNA template (20–200 ng). The PCR cycle was initial denaturation at 94°C for 3 min, followed by 35 cycles of denaturation at 94°C for 2 min, annealing at 58°C for 1 min and extension at 72°C for 3 min, with a final extension at 72°C for 7 min. All PCRs were performed in a Biometra TOne Thermal Cycler (Analytik Jena AG, Jena, Germany). The amplified products were analysed using 1.2% agarose gel electrophoresis. Purification of the PCR products was performed using a NucleoSpin^®^ Gel and PCR Clean-Up Kit (Macherey-Nagel, Germany) following the manufacturer's instructions. The purified PCR products were run on a 1.2% agarose gel at 100 V in 1× TBE buffer. The PCR products were sequenced in both the forward and reverse directions at Macrogen Inc., Seoul, Korea.

### 66-kDa protein sequences from GenBank

The nucleotide sequences of a 66-kDa protein-encoding gene of *A. cantonensis* and *A. malaysiensis* from Thailand, China, Japan, Malaysia and the United States downloaded from GenBank were included in the present study (Table S1). In addition, the sequences from *Ancylostoma caninum* and *Heterorhabditis bacteriophora* were used as outgroup taxa.

### Sequence and phylogenetic analysis

All nucleotide sequences were edited and assembled using Seq-Man II software (DNASTAR, Madison, WI, USA). Subsequently, multiple-sequence alignment with ClustalW and trimmed sequences was performed in MEGA version 7.0 (Kumar *et al*., 2016). The 66-kDa protein sequence was blasted in the GenBank database (http://blast.ncbi.nlm.nih.gov/Blast.cgi) to confirm species identification of *Angiostrongylus* (*A. cantonensis* and *A. malaysiensis*).

Phylogenetic trees were constructed *via* the maximum-likelihood (ML) and Bayesian inference (BI) methods. The ML tree with the Tamura–Nei model (Tamura and Nei, 1993) was generated *via* 1000 bootstrap replicates in MEGA version 7.0 (Kumar *et al*., 2016). The BI tree was constructed using the MrBayes 3.2.0 program (Ronquist *et al*., 2012). The Bayesian posterior probabilities (BPPs) were estimated using Markov chain Monte Carlo analysis, which was run for 10 000 000 generations with data sampling every 500 generations, discarding the first 1000 sampled trees as burn-in (Monte *et al*., 2012). The final phylogenetic trees were viewed and edited in FigTree v.1.4. The nucleotide variation and *P* distance of *A. cantonensis* and *A. malaysiensis* were calculated using the resultant alignment in MEGA version 7.0.

### Genetic analysis

The sequences of a 66-kDa protein-encoding gene obtained in the present study together with the sequences downloaded from GenBank were grouped into 3 datasets to analyse the genetic population and the transmission relationships between the host and parasite. Group 1 included all sequences of *A. cantonensis* (*n* = 53) and *A. malaysiensis* (*n* = 64) populations from different countries. Group 2 consisted of all sequences of *A. cantonensis* (*n* = 32) and *A. malaysiensis* (*n* = 60) from different regions of Thailand, and group 3 included all currently available sequences of *A. cantonensis* and *A. malaysiensis* isolated from intermediate land snail hosts and definitive rodent hosts from different countries.

The genealogical relationships were estimated by using a haplotype network constructed in Network 5.0.1.1 (http://www.fluxus-engineering.com) based on the median-joining algorithm (Bandelt *et al*., 1999). Apart from the *A. cantonensis* and *A. malaysiensis* 66-kDa protein-encoding sequences in this study, we also included the nucleotide sequences of these worms deposited in GenBank by Eamsobhana *et al*. (2019). The haplotype nomenclature used in this study was the same as that used by Eamsobhana *et al*. (2019).

Genetic diversity indices, e.g. haplotype number, segregating sites, haplotype diversity and nucleotide diversity, were computed and generated by DnaSp version 5 (Librado and Rozas, 2009) and ARLEQUIN version 3.5.1.2 (Excoffier and Lischer, 2010). Genetic differentiation between *Angiostrongylus* from different rodent host species was investigated by comparing genetic divergence within rodent host species based on the K2P model in MEGA version 7.0 (Kumar *et al*., 2016).

## Results

### Molecular identification of *Angiostrongylus* spp.

The molecular identification of *Angiostrongylus* spp. based on the 66-kDa protein gene was congruent with the morphological identification. PCR-based analysis and sequencing of the 66-kDa protein gene were performed together with a BLASTN search. Fourteen samples (245 bp) of *Angiostrongylus* (GenBank accession nos. OM280392–OM280405) were identified as *A. cantonensis* with the highest similarity (100%) with GenBank accession no. MH562093. In addition, 41 sequences of *A. malaysiensis* in this study (GenBank accession nos. OM280406–OM280446) showed 99–100% identity to *A. malaysiensis* (GenBank accession nos. MH562059 and MH562113) after a BLASTN search using 245 bp of the 66-kDa protein gene.

### Phylogenetic analyses

**Fig. 2.**
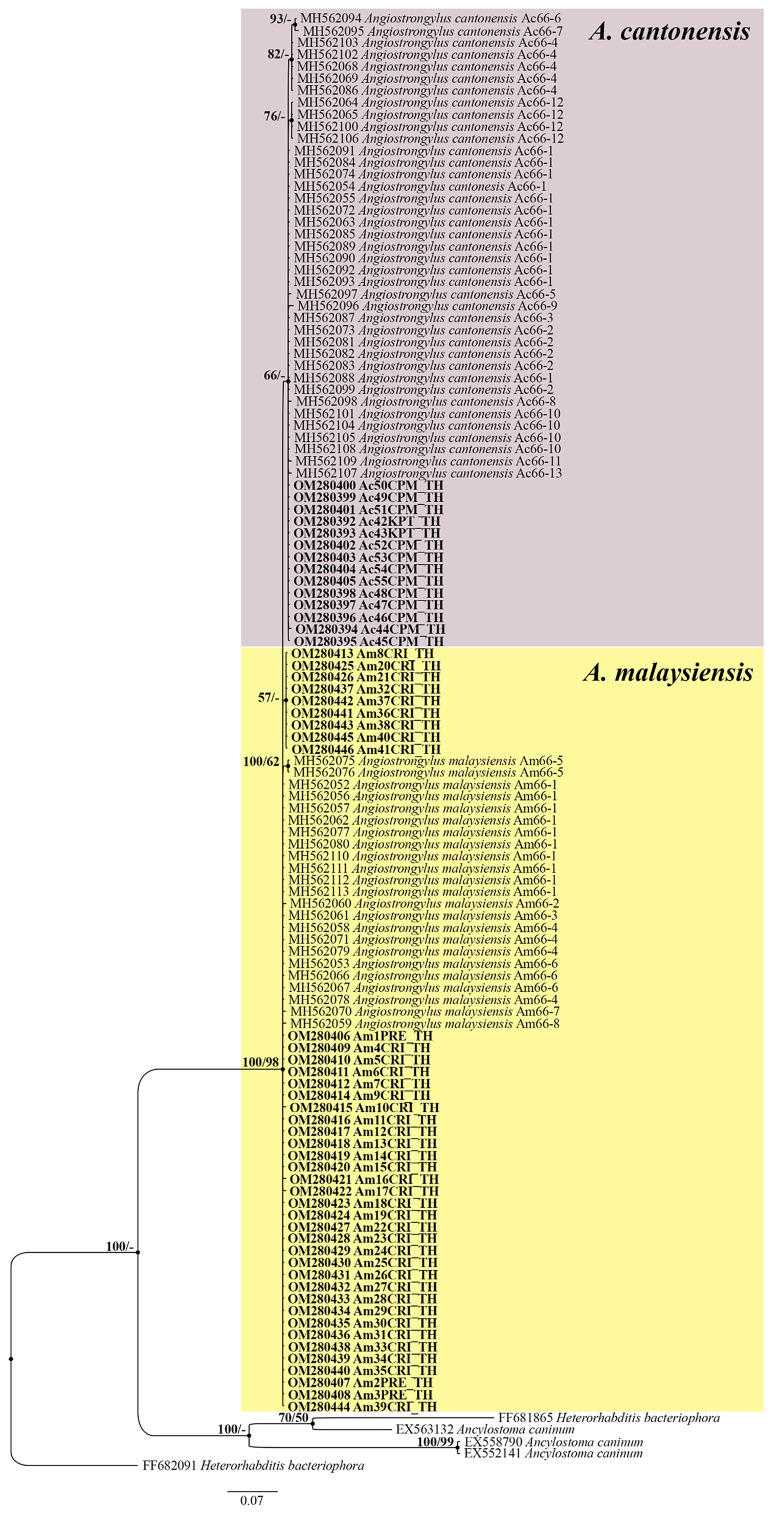
Bayesian tree of *A. cantonensis* and *A. malaysiensis* based on 66-kDa protein sequences (245 bp). The BPPs (left) and ML bootstrap values (right) are represented at each node of the phylogenetic tree. Bold letters indicate the sequences obtained in this study. *Ancylostoma caninum* and *Heterorhabditis bacteriophora* were used as the outgroup. Ac, *A. cantonensis*; Am, *A. malaysiensis*; CPM, Chaiyaphum; CRI, Chiang Rai; KPT, Kamphaeng Phet; PRE, Phrae; TH, Thailand.

The phylogenetic trees of *A. cantonensis* (53 sequences) and *A. malaysiensis* (64 sequences) were reconstructed using the BI and ML methods. Both methods revealed congruent topologies. Therefore, here we show the BI tree with posterior probabilities and only the bootstrap values from ML analyses. The phylogenetic trees based on 245 bp of the 66-kDa protein gene showed that *Angiostrongylus* formed a monophyletic clade with a clear separation between *A. cantonensis* and *A. malaysiensis* (Fig. 2). The interspecific distances between the *A. cantonensis* and *A. malaysiensis* sequences ranged from 0.82 to 2.86%, with a total of 16 variable sites found (Table 1).

**Table 1.** Haplotypes and their variable nucleotide positions in the 66-kDa protein gene of *Angiostrongylus cantonensis* and *Angiostrongylus malaysiensis*

Haplotype	Nucleotide sequence															
			1	1	1	1	1	1	1	1	2	2	2	2	2	2
	2	8	3	4	4	4	5	7	8	9	2	3	4	4	4	4
	3	5	5	0	5	7	7	5	0	0	6	0	1	3	4	5
*A. cantonensis* Ac66-1	A	A	A	T	T	G	G	C	G	C	G	T	A	G	A	T
*A. cantonensis* Ac66-2	A	A	A	T	T	G	G	C	G	C	G	T	A	G	T	T
*A. cantonensis* Ac66-3	A	A	A	T	T	G	G	C	G	C	G	T	A	G	T	–
*A. cantonensis* Ac66-4	A	A	A	A	T	G	G	C	G	C	G	T	A	G	A	T
*A. cantonensis* Ac66-5	A	A	A	T	T	G	G	C	G	C	G	T	A	G	A	C
*A. cantonensis* Ac66-6	A	A	A	A	T	G	G	C	G	C	G	T	A	G	A	C
*A. cantonensis* Ac66-7	A	A	A	A	T	G	G	C	G	T	G	T	A	G	A	C
*A. cantonensis* Ac66-8	A	A	A	T	T	G	G	C	A	C	G	T	A	G	A	C
*A. cantonensis* Ac66-9	A	A	A	T	T	G	G	C	G	C	G	G	A	G	C	–
*A. cantonensis* Ac66-10	A	A	A	T	T	G	G	C	A	C	G	T	A	G	A	T
*A. cantonensis* Ac66-11	A	A	A	T	T	G	G	C	A	C	G	T	T	G	A	T
*A. cantonensis* Ac66-12	A	A	A	T	T	G	G	C	A	C	G	T	A	G	T	T
*A. cantonensis* Ac66-13	A	A	A	T	T	G	G	C	A	C	C	T	A	G	A	T
***A. cantonensis* Ac66-14**	A	A	A	T	T	G	G	C	G	C	G	T	A	G	T	G
*A. malaysiensis* Am66-1	A	A	A	T	G	G	G	A	A	C	G	T	A	G	A	T
*A. malaysiensis* Am66-2	C	A	A	T	G	G	G	A	A	C	G	T	A	G	A	T
*A. malaysiensis* Am66-3	A	A	A	T	G	G	G	A	A	C	G	T	A	G	T	–
*A. malaysiensis* Am66-4	A	A	A	T	G	G	G	A	A	C	G	T	A	G	T	T
*A. malaysiensis* Am66-5	A	A	A	T	G	G	G	A	A	C	G	T	A	A	T	G
*A. malaysiensis* Am66-6	A	A	A	T	G	G	G	A	A	C	G	T	A	G	T	A
*A. malaysiensis* Am66-7	A	A	A	T	G	G	G	A	A	C	G	T	A	G	A	A
*A. malaysiensis* Am66-8	A	A	A	T	G	A	G	A	A	C	G	T	A	G	A	C
***A. malaysiensis* Am66-9**	A	A	A	T	G	A	G	A	A	C	G	T	A	G	A	T
***A. malaysiensis* Am66-10**	A	A	A	T	G	A	G	A	A	C	G	T	A	G	T	T
***A. malaysiensis* Am66-11**	A	A	G	T	G	G	G	A	A	C	G	T	A	G	A	T
***A. malaysiensis* Am66-12**	A	T	A	T	G	A	G	A	A	C	G	T	A	G	A	T
***A. malaysiensis* Am66-13**	A	A	A	T	G	A	A	A	A	C	G	T	A	G	A	T

Bold letters indicate the new haplotypes obtained in the present study.

### Genetic variation of *A. cantonensis* and *A. malaysiensis* populations from different countries

**Fig. 3.**
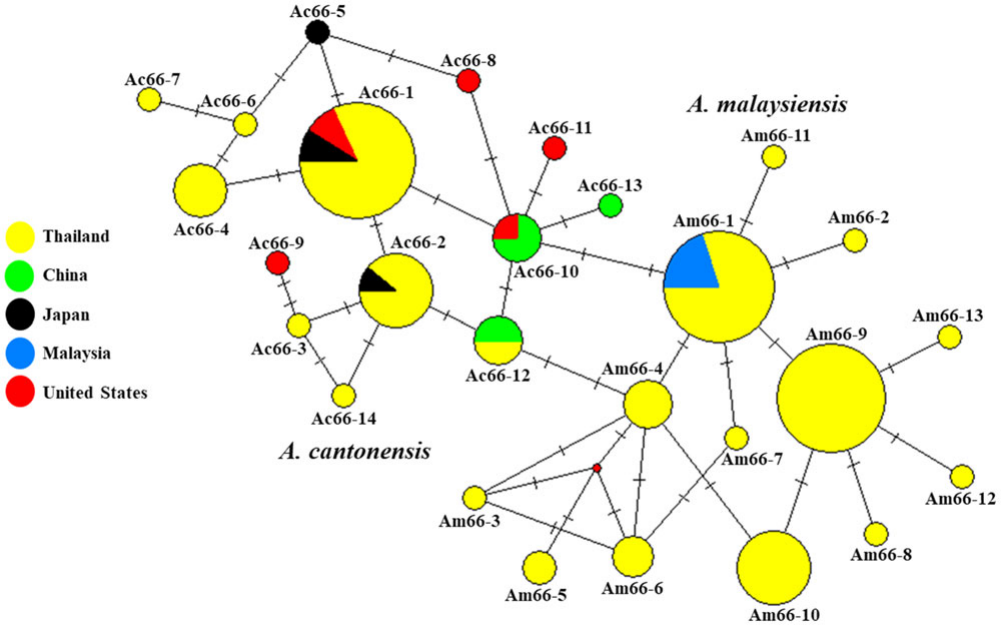
Median-joining haplotype networks of *A. cantonensis* and *A. malaysiensis* from Thailand and other geographical regions inferred from 66-kDa protein sequences. Each haplotype is represented by a circle, and circle sizes are proportional to haplotype frequency. Colours indicate the geographic origin of the haplotypes. Each mutation between haplotypes is represented by a bar. Median vectors (small red dots) represent ancestral haplotypes that are either not sampled or missing haplotypes.

Based on previous studies of *A. cantonensis*, 13 haplotypes (Ac66-1 to Ac66-13) were classified, and sequences were deposited in GenBank, i.e. haplotypes Ac66-1 to Ac66-4, and Ac66-12 consisted of sequences covering Thailand, China, Japan and the United States, and haplotypes Ac66-8 to Ac66-11 were found only in the United States. In the current haplotype network analyses, our 14 sequences together with 39 sequences from previous studies retrieved from GenBank revealed 14 haplotypes (Ac66-1 to Ac66-14) (Fig. 3). Among the 14 sequence samples obtained in the present study, 9 and 4 sequences belonged to haplotypes Ac66-1 and Ac66-2, respectively. In addition, 1 sequence obtained in the present study was identified as a new haplotype named Ac66-14. A comparison of nucleotide sequences between this new haplotype and 13 previously reported haplotypes is presented in Table 1. The genetic distances among the haplotypes varied from 0 to 0.016 (Table 2). Of these, 10 haplotypes were unique (Ac66-3 to Ac66-9, Ac66-11, Ac66-13 and Ac66-14), and 4 were shared by at least 2 populations (Ac66-1, Ac66-2, Ac66-10 and Ac66-12). The most widely distributed haplotype, Ac66-1, was shared among samples from Thailand, Japan and the United States. Haplotype Ac66-2 was shared between samples from Thailand and Japan. Haplotype Ac66-10 was shared between samples from China and the United States. Haplotype Ac66-12 was shared between samples from Thailand and China. The haplotype diversity in each population ranged from 0.7117 in Thailand to 0.9333 in the United States, with an average of 0.7903. The nucleotide diversity in each population ranged from 0.0035 in China to 0.0076 in the United States, with an average of 0.0054 (Table 3).

**Table 2.** Genetic distance among haplotypes of *A. cantonensis* based on 66-kDa protein sequences

Haplotypes	Ac66-1	Ac66-2	Ac66-3	Ac66-4	Ac66-5	Ac66-6	Ac66-7	Ac66-8	Ac66-9	Ac66-10	Ac66-11	Ac66-12	Ac66-13	Ac66-14
Ac66-1	–													
Ac66-2	0.004	–												
Ac66-3	0.004	0.000	–											
Ac66-4	0.004	0.008	0.008	–										
Ac66-5	0.000	0.004	0.004	0.004	–									
Ac66-6	0.004	0.008	0.008	0.000	0.004	–								
Ac66-7	0.008	0.012	0.012	0.004	0.008	0.004	–							
Ac66-8	0.004	0.008	0.008	0.008	0.004	0.008	0.012	–						
Ac66-9	0.008	0.008	0.008	0.012	0.008	0.012	0.016	0.012	–					
Ac66-10	0.004	0.008	0.008	0.008	0.004	0.008	0.012	0.000	0.012	–				
Ac66-11	0.008	0.012	0.012	0.012	0.008	0.012	0.016	0.004	0.016	0.004	–			
Ac66-12	0.008	0.004	0.004	0.012	0.008	0.012	0.016	0.004	0.012	0.004	0.008	–		
Ac66-13	0.008	0.012	0.012	0.012	0.008	0.012	0.016	0.004	0.016	0.004	0.008	0.008	–	
Ac66-14	0.004	0.000	0.000	0.008	0.004	0.008	0.012	0.008	0.008	0.008	0.012	0.004	0.012	–

**Table 3.** Genetic diversity of *A. cantonensis* and *A. malaysiensis* from Thailand and other geographical regions based on 66-kDa protein sequences

Species	Countries	Haplotypes			Samples				Haplotype diversity (*h*), mean ± s.d.	Nucleotide diversity (*π*), mean ± s.d.
		Number of haplotypes	Shared haplotypes	Unique haplotypes	Haplotype name	Number of samples	Eamsobhana *et al*. (2019)	Present study		
*A. cantonensis*	Thailand	8	3	5	Ac66-1	18	9	9	0.7117 ± 0.0619	0.0044 ± 0.0033
					Ac66-2	8	4	4		
					Ac66-3	1	1	0		
					Ac66-4	5	5	0		
					Ac66-6	1	1	0		
					Ac66-7	1	1	0		
					Ac66-12	2	2	0		
					Ac66-14	1	0	1		
	China	3	2	1	Ac66-10	3	3	0	0.7333 ± 0.1552	0.0035 ± 0.0033
					Ac66-12	2	2	0		
					Ac66-13	1	1	0		
	Japan	3	2	1	Ac66-1	2	2	0	0.8333 ± 0.2224	0.0041 ± 0.0040
					Ac66-2	1	1	0		
					Ac66-5	1	1	0		
	United States	5	2	3	Ac66-1	2	2	0	0.9333 ± 0.1217	0.0076 ± 0.0058
					Ac66-9	1	1	0		
					Ac66-8	1	1	0		
					Ac66-10	1	1	0		
					Ac66-11	1	1	0		
	Total	14	4	10		53	39	14	0.7903 ± 0.0467	0.0054 ± 0.0038
*A. malaysiensis*	Thailand	13	1	12	Am66-1	16	6	10	0.8096 ± 0.0307	0.0056 ± 0.0039
					Am66-2	1	1	0		
					Am66-3	1	1	0		
					Am66-4	4	4	0		
					Am66-5	2	2	0		
					Am66-6	3	3	0		
					Am66-7	1	1	0		
					Am66-8	1	1	0		
					Am66-9	19	0	19		
					Am66-10	9	0	9		
					Am66-11	1	0	1		
					Am66-12	1	0	1		
					Am66-13	1	0	1		
	Malaysia	1	1	0	Am66-1	4	4	0	0	0
	Total	13	1	12		64	23	41	0.7981 ± 0.0307	0.0054 ± 0.0038

Thirteen haplotypes (Am66-1 to Am66-13) of *A. malaysiensis* were identified from 41 sequences obtained in the present study together with 23 sequences from GenBank. Among the 41 samples from the present study, 10 sequences belonged to haplotype Am66-1, and 31 sequences belonged to the 5 new haplotypes, including Am66-9 (19 sequences), Am66-10 (9 sequences), Am66-11 (1 sequence), Am66-12 (1 sequence) and Am66-13 (1 sequence). A comparison of nucleotide sequences between the 5 new haplotypes identified in the present study and 8 previously reported haplotypes is presented in Table 1. The genetic distances between the haplotypes varied from 0 to 0.016 (Table 4). Of these, 12 haplotypes were unique (Am66-2 to Am66-13), and 1 (Am66-1) was shared between samples from Malaysia and Thailand (Fig. 3). The haplotype diversity in each population ranged from 0 in Malaysia to 0.8096 in Thailand, with an average of 0.7981. The nucleotide diversity in each population ranged from 0 in Malaysia to 0.0056 in Thailand, with an average of 0.0054 (Table 3).

**Table 4.** Genetic distance among haplotypes of *A. malaysiensis* based on 66-kDa protein sequences

Haplotypes	Am66-1	Am66-2	Am66-3	Am66-4	Am66-5	Am66-6	Am66-7	Am66-8	Am66-9	Am66-10	Am66-11	Am66-12	Am66-13
Am66-1	–												
Am66-2	0.004	–											
Am66-3	0.004	0.008	–										
Am66-4	0.004	0.008	0.000	–									
Am66-5	0.008	0.012	0.004	0.004	–								
Am66-6	0.004	0.008	0.000	0.000	0.004	–							
Am66-7	0.000	0.004	0.004	0.004	0.008	0.004	–						
Am66-8	0.004	0.008	0.008	0.008	0.012	0.008	0.004	–					
Am66-9	0.004	0.008	0.008	0.008	0.012	0.008	0.004	0.000	–				
Am66-10	0.008	0.012	0.004	0.004	0.008	0.004	0.008	0.004	0.004	–			
Am66-11	0.004	0.008	0.008	0.008	0.012	0.008	0.004	0.008	0.008	0.012	–		
Am66-12	0.008	0.012	0.012	0.012	0.016	0.012	0.008	0.004	0.004	0.008	0.012	–	
Am66-13	0.008	0.012	0.012	0.012	0.016	0.012	0.008	0.004	0.004	0.008	0.012	0.008	–

### Genetic variation of *A. cantonensis* and *A. malaysiensis* isolated from different regions of Thailand

**Fig. 4.**
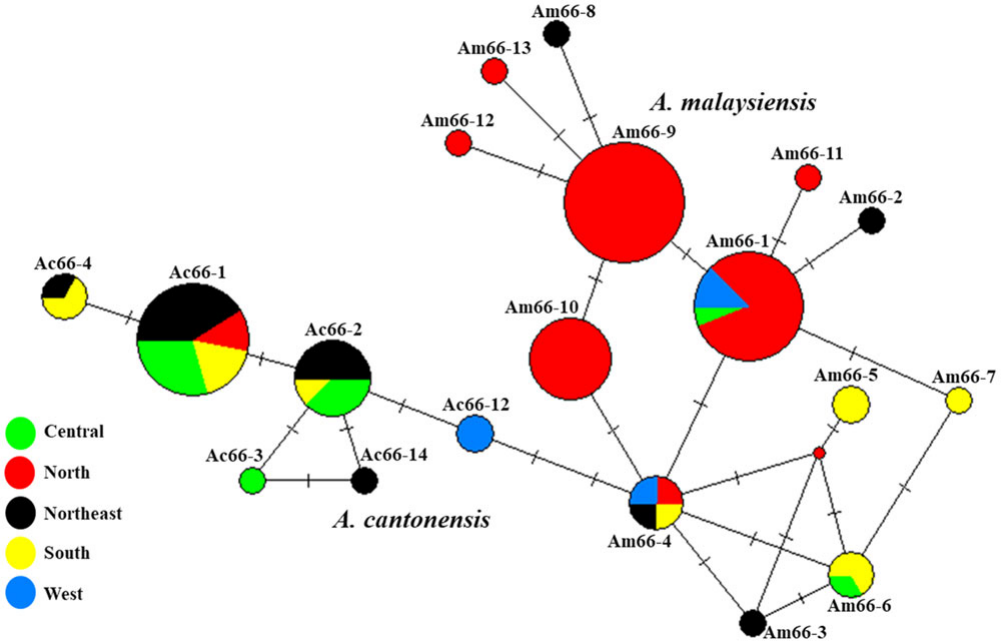
Median-joining haplotype networks of *A. cantonensis* and *A. malaysiensis* from different regions of Thailand inferred from 66-kDa protein sequences. Each haplotype is represented by a circle, and circle sizes are proportional to haplotype frequency. Colours indicate the geographic origin of the haplotypes. Each mutation between haplotypes is represented by a bar. Median vectors (small red dots) represent ancestral haplotypes that are either not sampled or missing haplotypes.

The analysis of 32 *A. cantonensis* sequences from Thailand identified 6 haplotypes (Ac66-1 to Ac66-4, Ac66-12 and Ac66-14). Among the *A. cantonensis* haplotypes in Thailand, Ac66-1 was the most common and was widely distributed in 4 regions (central, north, northeast and south). Another common haplotype was Ac66-2, which was found in several geographical localities in 3 regions (central, northeast and south), and haplotype Ac66-4 was found in the northeast and south regions of Thailand. The remaining haplotypes were found to be unique in a particular isolate, such as haplotype Ac66-3, which was found only in the central isolate, Ac66-12, which was unique to the western isolate, and Ac66-14, which was found only in the northeast isolate (Fig. 4). The haplotype diversity in each population ranged from 0 in the population from the north and west regions to 0.7333 in the population from the south, with an average of 0.6613. The nucleotide diversity in each population ranged from 0 in the north and west regions to 0.8205 in the northeast region, with an average of 0.0034 (Table 5).

**Table 5.** Genetic diversity of *A. cantonensis* and *A. malaysiensis* from different regions of Thailand based on 66-kDa protein sequences

Species	Regions	Haplotypes	Samples	Haplotype diversity (*h*), mean ± s.d.	Nucleotide diversity (*π*) mean ± s.d.						
		Number of haplotypes	Shared haplotypes	Unique haplotypes	Haplotype name	Number of samples	Eamsobhana *et al*. (2019)	Present study				
*A. cantonensis*	Central	3	2	1	Ac66-1	5	3	2		0.6389 ± 0.1258	0.0023 ± 0.0023
					Ac66-2	3	3	0			
					Ac66-3	1	1	0			
	North	1	1	0	Ac66-1	2	2	0	0		0
	Northeast	4	3	1	Ac66-1	7	0	7		0.6538 ± 0.1060	0.8205 ± 0.6269
					Ac66-2	4	0	4			
					Ac66-4	1	1	0			
					Ac66-14	1	0	1			
	South	3	3	0	Ac66-1	3	3	0		0.7333 ± 0.1552	0.0035 ± 0.0033
					Ac66-2	1	1	0			
					Ac66-4	2	2	0			
	West	1	0	1	Ac66-12	2	2	0		0	0
	Total	6	3	3		32	18	14		0.6613 ± 0.0707	0.0034 ± 0.0028
*A. malaysiensis*	Central	2	2	0	Am66-1	1	1	0		1.0000 ± 0.5000	0.0082 ± 0.0099
					Am66-6	1	1	0			
	North	7	2	5	Am66-1	13	3	10		0.7121 ± 0.0392	0.0038 ± 0.0029
					Am66-4	1	1	0			
					Am66-9	19	0	19			
					Am66-10	9	0	9			
					Am66-11	1	0	1			
					Am66-12	1	0	1			
					Am66-13	1	0	1			
	Northeast	4	1	3	Am66-2	1	1	0		1.0000 ± 0.1768	0.0082 ± 0.0069
					Am66-3	1	1	0			
					Am66-4	1	1	0			
					Am66-8	1	1	0			
	South	4	2	2	Am66-4	1	1	0		0.8667 ± 0.1291	0.0065 ± 0.0052
					Am66-5	2	2	0			
					Am66-6	2	2	0			
					Am66-7	1	1	0			
	West	2	2	0	Am66-1	2	2	0		0.6667 ± 0.3143	0.0027 ± 0.0034
					Am66-4	1	1	0			
	Total	13	3	10		60	19	41		0.8096 ± 0.0307	0.0056 ± 0.0039

Thirteen haplotypes (Am66-1 to Am66-13) of *A. malaysiensis* were identified from 60 Thailand sequences. Among the *A. malaysiensis* haplotypes, Am66-4 was the most common and was widely distributed in 4 regions (north, northeast, south and west). Another common haplotype was Am66-1, which was found in several geographical localities in 3 regions (central, north and west), and haplotype Am66-6 was found in isolates from the central and south regions of Thailand. The remaining haplotypes were found to be unique in a particular isolate, such as haplotypes Am66-2, Am66-3 and Am66-8, which were found only in the northeast isolate; Am66-5 and Am66-7, which were unique to the south isolate and Am66-9 to Am66-13, which were present only in the north isolate (Fig. 4). The haplotype diversity in each population ranged from 0.6667 in the population from the west region to 1.0000 in the population from the central and northeast regions, with an average of 0.8096. The nucleotide diversity in each population ranged from 0.0027 in the west region to 0.0082 in the central and northeast regions, with an average of 0.0056 (Table 5).

### Genetic variation of *A. cantonensis* and *A. malaysiensis* isolated from snail intermediate and rodent definitive hosts

**Fig. 5.**
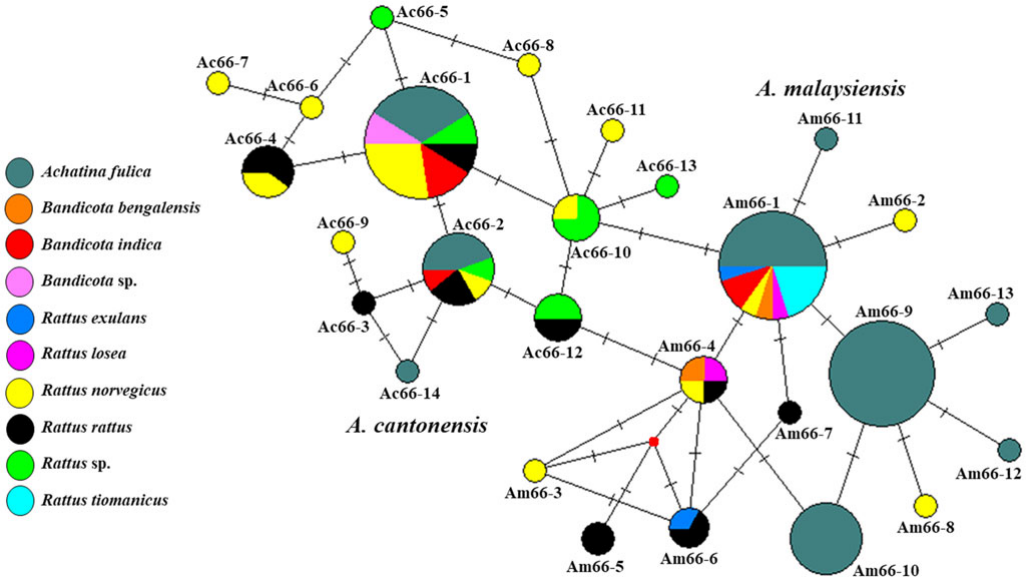
Median-joining haplotype networks of *A. cantonensis* and *A. malaysiensis* from different land snail and rodent host species inferred from 66-kDa protein sequences. Each haplotype is represented by a circle, and circle sizes are proportional to haplotype frequency. Colours indicate the geographic origin of the haplotypes. Each mutation between haplotypes is represented by a bar. Median vectors (small red dots) represent ancestral haplotypes that are either not sampled or missing haplotypes.

This group consisted of 53 and 64 sequences that were isolated from intermediate land snail hosts and definitive rodent hosts, respectively. In the land snail *A. fulica*, 12 *A. cantonensis* and 41 *A. malaysiensis* sequences were classified into 3 haplotypes (Ac66-1, Ac66-2 and Ac66-14) and 6 haplotypes (Am66-1, Am66-9 to Am66-13), respectively (Fig. 5). Among the *A. cantonensis* haplotypes, 3 haplotypes (Ac66-1, Ac66-2 and Ac66-14) were found in *A. fulica* in Chaiyaphum province of Thailand. Of these, only 2 haplotypes (Ac66-1 and Ac66-2) were found in rodent hosts from different countries, such as haplotype Ac66-1 found in isolates from Thailand, Japan and the United States, and Ac66-2 was present in isolates from Thailand and Japan. In *A. malaysiensis*, Am66-1 was the most widely distributed in *A. fulica* in 2 provinces (Chiang Rai and Phrae) in northern Thailand. In addition, this haplotype was found in rodent hosts from the central province (Bangkok), 2 northern provinces (Chiang Rai and Mae Hong Son) and 2 western provinces (Kanchanaburi and Tak) of Thailand and in Pahang in Malaysia.

For the rodent host species, the haplotype diversity of *A. cantonensis* ranged from 0 in *Bandicota* sp. to 0.8889 in *Rattus* species. Nucleotide diversity ranged from 0 in *Bandicota* sp. to 0.0071 in *Rattus norvegicus*. Among the 13 haplotypes identified, 8 were unique, and 5 haplotypes were shared by at least 2 rodent host species. *Rattus norvegicus* possessed the highest number (5 haplotypes) of unique haplotypes (Fig. 5, Table 6). Genetic divergence within the rodent host species based on the K2P model ranged from 0 to 1.66%, with a mean of 0.47% (Table 6). The greatest within-rodent host genetic divergence (1.66%) was found in *R. norvegicus*. In *A. malaysiensis*, the haplotype diversity ranged from 0 in *Bandicota indica* and *Rattus tiomanicus* to 1.0000 in *Bandicota bengalensis*, *Rattus exulans*, *Rattus losea* and *Rattus norvegicus*. Nucleotide diversity ranged from 0 in *B. indica* and *R. tiomanicus* to 0.0082 in *R. exulans*. Among the 8 haplotypes identified, 5 were unique, and 3 haplotypes were shared by at least 2 rodent host species. *Rattus norvegicus* possessed the highest number (3 haplotypes) of unique haplotypes (Fig. 5, Table 6). Genetic divergence within the rodent host species based on the K2P model ranged from 0 to 1.24%, with a mean of 0.41% (Table 6). The greatest within-rodent host genetic divergence (1.24%) was found in *R. rattus*.

**Table 6.** Haplotype diversity (*h*), nucleotide diversity (*h*) and genetic divergence based on 66-kDa sequences between *A. cantonensis* and *A. malaysiensis* from different host species

Species	Host species	Number of sampling location	Haplotypes			Samples				Haplotype diversity (*h*), mean ± s.d.	Nucleotide diversity (*π*), mean ± s.d.	Per cent genetic divergences within host species (mean)
			Number of haplotypes	Shared haplotypes	Unique haplotypes	Haplotype name	Number of samples	Eamsobhana *et al*. (2019)	Present study			
*A. cantonensis*	*Achatina fulica*	1	3	2	1	Ac66-1	7	0	7	NA	NA	NA
						Ac66-2	4	0	4			
						Ac66-14	1	0	1			
	*Bandicota indica*	2	2	2	0	Ac66-1	3	3	0	0.5000 ± 0.2652	0.0020 ± 0.0025	0–0.41 (0.21)
						Ac66-2	1	1	0			
	*Bandicota* sp.	1	1	1	0	Ac66-1	2	0	2	0	0	0 (0)
	*Rattus norvegicus*	4	9	4	5	Ac66-1	6	6	0	0.8476 ± 0.0878	0.0071 ± 0.0049	0–1.66 (0.59)
						Ac66-2	1	1	0			
						Ac66-4	2	2	0			
						Ac66-6	1	1	0			
						Ac66-7	1	1	0			
						Ac66-8	1	1	0			
						Ac66-9	1	1	0			
						Ac66-10	1	1	0			
						Ac66-11	1	1	0			
	*Rattus rattus*	6	5	4	1	Ac66-1	2	2	0	0.8667 ± 0.0714	0.0056 ± 0.0043	0–1.24 (0.57)
						Ac66-2	2	2	0			
						Ac66-3	1	1	0			
						Ac66-4	3	3	0			
						Ac66-12	2	2	0			
	*Rattus* sp.	2	6	4	2	Ac66-1	2	2	0	0.8889 ± 0.0754	0.0057 ± 0.0043	0–1.24 (0.57)
						Ac66-2	1	1	0			
						Ac66-5	1	1	0			
						Ac66-10	3	3	0			
						Ac66-12	2	2	0			
						Ac66-13	1	1	0			
	Total	16	14	5	9		53	39	14	0.8329 ± 0.0451	0.0060 ± 0.0041	0–1.66 (0.47)
*A. malaysiensis*	*A. fulica*	2	6	1	5	Am66-1	10	0	10	NA	NA	NA
						Am66-9	19	0	19			
						Am66-10	9	0	9			
						Am66-11	1	0	1			
						Am66-12	1	0	1			
						Am66-13	1	0	1			
	*Bandicota bengalensis*	1	2	2	0	Am66-1	1	1	0	1.0000 ± 0.5000	0.0041 ± 0.0058	0–0.41 (0.41)
						Am66-4	1	1	0			
	*B. indica*	1	1	1	0	Am66-1	2	2	0	0	0	0 (0)
	*Rattus exulans*	1	2	2	0	Am66-1	1	1	0	1.0000 ± 0.5000	0.0082 ± 0.0099	0–0.82 (0.82)
						Am66-6	1	1	0			
	*Rattus losea*	1	2	2	0	Am66-1	1	1	0	1.0000 ± 0.5000	0.0041 ± 0.0058	0–0.41 (0.41)
						Am66-4	1	1	0			
	*R. norvegicus*	2	5	2	3	Am66-1	1	1	0	1.0000 ± 0.1265	0.0069 ± 0.0056	0–0.83 (0.58)
						Am66-2	1	1	0			
						Am66-3	1	1	0			
						Am66-4	1	1	0			
						Am66-8	1	1	0			
	*R. rattus*	3	4	2	2	Am66-4	1	1	0	0.8667 ± 0.1291	0.0065 ± 0.0052	0–1.24 (0.66)
						Am66-5	2	2	0			
						Am66-6	2	2	0			
						Am66-7	1	1	0			
	*Rattus tiomanicus*	1	1	1	0	Am66-1	4	4	0	0	0	0 (0)
	Total	12	13	3	10		64	23	41	0.7826 ± 0.0728	0.0054 ± 0.0039	0–1.24 (0.41)

NA, not analysed.

## Discussion

The advantage of the 66-kDa protein gene as a genetic marker to determine genetic diversity and phylogeny between and within *Angiostrongylus* populations (*A. cantonensis*, *A. malaysiensis* and *A. costaricensis*) was previously noted (Eamsobhana *et al*., 2010, 2019). In this study, identification of *Angiostrongylus* based on a partial sequence of a 66-kDa protein-encoding gene was confirmed by 99–100% sequence identity after BLASTN searches. *Angiostrongylus cantonensis* and *A. malaysiensis* are closely related in terms of morphological and genetic characteristics and also share similarities in their life cycles (Chan *et al*., 2020; Watthanakulpanich *et al*., 2021). Both species utilize the same definitive and intermediate host species (Bhaibulaya and Techasoponmani, 1972). In addition, mixed infections with both *A. cantonensis* and *A. malaysiensis* have been widely recorded in snail intermediate hosts and rodent definitive hosts (Jakkul *et al*., 2021; Watthanakulpanich *et al*., 2021). The 66-kDa protein gene is undoubtedly suitable for discrimination between *A. cantonensis* and *A. malaysiensis* because the phylogenetic trees clearly place these 2 species into separate clades. Our findings are similar to those of previous reports (Eamsobhana *et al*., 2010, 2019). This suggests the advantage of the 66-kDa protein sequence for the identification of *A. cantonensis* and *A. malaysiensis*. Interestingly, the use of the 66-kDa protein gene as the genetic marker was successful in discriminating the third-stage larvae of *A. cantonensis* and *A. malaysiensis*.

The level of genetic divergence of the 66-kDa protein sequences between *A. cantonensis* and *A. malaysiensis* ranged from 0.82 to 2.86%, which was relatively low when compared with other genetic markers. Our findings are similar to those of Eamsobhana *et al*. (2019), who reported that the genetic distance between *A. cantonensis* and *A. malaysiensis* using 66-kDa protein sequences was approximately 3.27% (Eamsobhana *et al*., 2019). A lower genetic divergence (0–1.0%) in the nuclear 18S rRNA gene was also noted between *A. cantonensis* and *A. malaysiensis* (Chan *et al*., 2020). In contrast, the genetic divergence between *A. cantonensis* and *A. malaysiensis* was relatively high based on COI (9.8–16.4%), cytb (10.9–12.2%), 12S rRNA (6.8–7.9%), 16S rRNA (7.9–10.0%) and ITS2 (15.1–15.7%) sequences (Chan *et al*., 2020). However, several reports have shown that the nuclear 18S rRNA gene can be used to discriminate between *A. cantonensis* and *A. malaysiensis*, which are clearly distinguished into their clades using this marker despite the low interspecies genetic distance (Fontanilla and Wade, 2008; Tokiwa *et al*., 2012; Rodpai *et al*., 2016), which is comparable to the results we obtained using the 66-kDa protein gene.

Previous studies of third-stage larvae of *A. cantonensis* isolated from *A. fulica* snails in 8 provinces of Thailand using 8 random-amplified polymorphic DNA-PCR markers revealed high levels of genetic diversity and low levels of gene flow in *A. cantonensis* populations (Thaenkham *et al*., 2012). The worms from these 8 localities were divided into 2 groups with statistically significant genetic differentiation of the 2 populations: group 1 contained *A. cantonensis* from Chanthaburi, Chiang Mai, Khon Kaen, Narathiwat Nong Khai and Prachuap Khiri Khan provinces, and group 2 contained *A. cantonensis* from Kanchanaburi and Lop Buri provinces. Similarly, Vitta *et al*. (2016) reported third-stage larvae of *A. cantonensis* from freshwater and land snails in 19 distinct geographical areas of Thailand using COI sequences, revealing 2 different origins of *A. cantonensis* in Thailand: group 1 contained *A. cantonensis* from Kamphaeng Phet, Phetchabun, Tak and Thailand ac4, and group 2 contained *A. cantonensis* from Kalasin, Kamphaeng Phet, Phitsanulok, Tak and AC Thai. Nonetheless, haplotypes of groups 1 and 2 were found in the same areas (Kamphaeng Phet and Tak provinces). The results indicate the occurrence of restricted gene flow between localities.

A recent study reported the presence of 13 distinct 66-kDa protein sequence haplotypes (Ac66-1 to Ac66-13) from *A. cantonensis* in several parts of the world (Eamsobhana *et al*., 2019). Haplotype Ac66-1 was the most common haplotype in *A. cantonensis* from Thailand, Japan and the United States. Haplotype Ac66-5 was reported in Japan, while haplotypes Ac66-8, Ac66-9 and Ac66-11 were reported in the United States. Haplotype Ac66-10 was reported in China and the United States, and haplotype Ac66-13 was found only in China. In Thailand, *A. cantonensis* did not cluster unequivocally according to their geographical origin, as 7 haplotypes (Ac66-1 to Ac66-4, Ac66-6, Ac66-7 and Ac66-12) from 10 geographical regions of Thailand were found. The *A. cantonensis* haplotypes from Bangkok and Phitsanulok provinces in the central region, Surat Thani province in the south, and the Thailand laboratory strain (originating from Khon Kaen in the northeast) were variable. Moreover, 4 haplotypes were found confined to a single locality: Ac66-3 in Phitsanulok province (central region); Ac66-6 and Ac66-7 in the Thailand laboratory strain and Ac66-12 in Prachuap Khiri Khan province (west region) (Eamsobhana *et al*., 2019). In the present study, haplotype Ac66-1 was found in Kamphaeng Phet province (2 specimens) in the central region and Chaiyaphum province (7 specimens) in the northeast region of Thailand. Additionally, haplotype Ac66-1 was previously reported in 3 central provinces (Bangkok, Lop Buri and Phitsanulok), 2 southern provinces (Ranong and Surat Thani) and in the northern province (Chiang Mai) (Eamsobhana *et al*., 2019). This Ac66-1 haplotype was dominant in Thailand. The other 4 specimens (haplotype Ac66-2) from Chaiyaphum province in the present study were also reported from 2 central provinces (Bangkok and Samut Prakan) and the south province (Surat Thani). Importantly, 1 new haplotype (Ac66-14) was identified in 1 specimen from Chaiyaphum province of Thailand. Incorporation of the genetic data of the 66-kDa protein gene from *A. cantonensis* obtained in the present and previous studies (Eamsobhana *et al*., 2019) revealed sharing of dominant haplotypes (Ac66-1 and Ac66-2), suggesting a common origin.

Based on the 66-kDa protein sequence from *A. malaysiensis*, previous studies reported 8 haplotypes (Am66-1 to Am66-8) in Thailand and Malaysia (Eamsobhana *et al*., 2019). In this study, 13 haplotypes were identified, with 5 haplotypes being new. One haplotype (Am66-1) of *A. malaysiensis* from Phrae (3 specimens) and Chiang Rai (7 specimens) provinces in the north region was previously reported from Thailand and Malaysia (Eamsobhana *et al*., 2019). Five new haplotypes (Am66-9, Am66-10, Am66-11, Am66-12 and Am66-13) were reported from 31 specimens in Chiang Rai province of Thailand. The most common haplotype detected in Thailand and Malaysia was Am66-1. In Thailand, 66-kDa protein haplotypes (Am66-1 to Am66-8) were distributed at random throughout the country, and Am66-1 was the most widely distributed in 2 northern provinces (Chiang Rai and Mae Hong Son), 2 western provinces (Kanchanaburi and Tak) and in a central province (Bangkok). In addition, the *A. malaysiensis* haplotypes from Bangkok in central Thailand, Mae Hong Son in north Thailand, Nong Khai in northeast Thailand, Satun in south Thailand and Tak in west Thailand showed variable 66-kDa haplotype diversity. Moreover, 5 haplotypes were found confined to a single locality: Am66-2, Am66-3 and Am66-8 in Nong Khai province (northeast region); Am66-5 in Phang Nga province (south region) and Am66-7 in Satun province (south region) (Eamsobhana *et al*., 2019). Therefore, it was difficult to conclude the relationship between the haplotype of *A. malaysiensis* and geographic areas in Thailand. A larger sample size of the 66-kDa protein-encoding gene sequence may reveal a clearer relationship between haplotypes and localization in Thailand.

High haplotype diversity in a 66-kDa protein gene for *A. cantonensis* (14 haplotypes) and *A. malaysiensis* (13 haplotypes) has also been observed in other genetic markers. Twenty cytb haplotypes have been reported for *A. cantonensis* from several parts of the world (Dusitsittipon *et al*., 2015; Yong *et al*., 2015; Dumidae *et al*., 2019). For the COI gene, 16 haplotypes were observed in *A. cantonensis* globally (Eamsobhana *et al*., 2017), and 9 haplotypes were observed in *A. malaysiensis* from Laos, Malaysia, Myanmar and Thailand (Rodpai *et al*., 2016; Eamsobhana *et al*., 2018). For the 12S rRNA gene of *Angiostrongylus* in Thailand, the average genetic variation was 0.5% with 6 haplotypes in a population of *A. cantonensis* (Chan *et al*., 2020). Similarly, using the 16S rRNA gene, an average genetic variation of 2.2% with 6 haplotypes within 1 population of *A. cantonensis* and an average genetic variation of 1.7% with 6 haplotypes within 4 populations of *A. malaysiensis* were found (Chan *et al*., 2020). Comparatively, we found that the 66-kDa protein gene resulted in 3 haplotypes within 2 populations of *A. cantonensis* (Chaiyaphum and Kamphaeng Phet provinces). Moreover, 6 haplotypes were found in 2 populations of *A. malaysiensis* (Phrae and Chiang Rai provinces). Higher intraspecific genetic variation levels and more haplotypes might be observed if more *A. cantonensis* and *A. malaysiensis* specimens are sampled from other localities.

To investigate the distribution of haplotypes in hosts, median-joining networks were constructed to analyse all currently available 66-kDa protein sequences of *A. cantonensis* and *A. malaysiensis* isolated from snail intermediate hosts (*A. fulica*) and rodent definitive hosts from different countries. In *A. cantonensis*, 2 haplotypes (Ac66-1 and Ac66-2) were shared between *A. cantonensis* isolated from *A. fulica* (from Chaiyaphum province of Thailand) and rodent hosts (from Thailand, Japan and the United States). In *A. malaysiensis*, Am66-1 was the most widely distributed haplotype in *A. fulica* in 2 northern provinces (Chiang Rai and Phrae) of Thailand. In addition, this haplotype was found in rodent hosts from the central province (Bangkok), 2 northern provinces (Chiang Rai and Mae Hong Son) and 2 western provinces (Kanchanaburi and Tak) of Thailand and in Pahang in Malaysia. This could be considered the result of range expansion of genomic origin. These findings indicate that lineage-specific *A. cantonensis* and *A. malaysiensis* have been spreading across Thailand. The transmission of this nematode has been linked to the dispersal of invasive hosts (Tokiwa *et al*., 2012). The increased presence of *A. cantonensis* in the country is likely a result of the rapid spread of its intermediate host, *A. fulica*, contributing to the dispersion of this parasite and infection of the definitive host (Thiengo *et al*., 2010; Dumidae *et al*., 2019, 2021). Invasion by *A. fulica* facilitates the establishment of the life cycle of the parasite and thus increases the chances for exposure of native snails to *A. cantonensis* in existing endemic areas. In addition, this invasive snail accelerates the spread of *A. cantonensis* to new areas since it rapidly expands the parasite's range (Lv *et al*., 2009). This phenomenon is described as one of the primary causes of the spread of oeosinophilic meningitis (Maldonado *et al*., 2010). Invasive snails and rodents are implicated in an increase in the distribution of *A. cantonensis* in Brazil (Thiengo *et al*., 2010), Spain (Foronda *et al*., 2010), China (Yang *et al*., 2013), Uganda (Mugisha *et al*., 2012), Japan (Tokiwa *et al*., 2013) and the United States (York *et al*., 2015). The hypothesis that *Angiostrongylus* has achieved global-scale dispersal with various organisms/hosts or vectors is largely influenced by human transportation (Monte *et al*., 2012; Tokiwa *et al*., 2012; Dusitsittipon *et al*., 2015).

Comparisons of genetic divergence among rodent host species found unique and shared haplotypes in different rodent species. All rodent host species included in the present study shared at least 1 common 66-kDa protein haplotype. Our findings showed that 8 haplotypes were unique and 5 haplotypes were shared in *A. cantonensis*, whereas 5 haplotypes of *A. malaysiensis* were found to be unique, and 3 haplotypes were shared by at least 2 rodent host species. The high degree of haplotype uniqueness suggests that there are some limitations to the spread of genomic origin among rodent host species. Many rodent species serve as definitive hosts for *A. cantonensis* and *A. malaysiensis* and are capable of highly promoting the distribution and intraspecific transfer of this parasite (Eamsobhana *et al*., 2016). The limited dispersal of rodent hosts might be expected to limit the genomic origin of their worm parasites, resulting in genetic structure over small geographical scales (Pocock *et al*., 2005; Gardner-Santana *et al*., 2009). However, data on the genetic diversity of *Angiostrongylus* remain scarce in invaded areas (Simões *et al*., 2011; Monte *et al*., 2012; Moreira *et al*., 2013; Dalton *et al*., 2017). The pathogenicity of *A. cantonensis* against laboratory hosts varies between different *A. cantonensis* genetic strains (Lee *et al*., 2014). Whether this dominant haplotype presents any difference in pathogenicity compared to other haplotypes remains to be clarified. Further studies are needed to clarify whether this haplotype has a different pathogenicity that may contribute to its evolution.

## Conclusion

This study further confirmed the presence of 66-kDa protein genetic diversity in various geographical isolates of *A. cantonensis* and *A. malaysiensis*. We demonstrate the utility of the 66-kDa protein-encoding gene as a genetic marker for species discrimination in the larval stage, as it clearly discriminated *A. cantonensis* and *A. malaysiensis* into separate clades. Importantly, we identified 1 and 5 new haplotypes from *A. cantonensis* and *A. malaysiensis*, respectively. Our findings revealed that the 66-kDa protein gene has sufficient intraspecific genetic variation to be considered a genetic marker for future *Angiostrongylus* population-level studies.

## Data Availability

All relevant data are within the paper and its supporting information file.

## Supplementary material

For supplementary material accompanying this paper visit https://doi.org/10.1017/S0031182022001573. click here to view supplementary material
