# Supplementary material for: Genetic analysis of a 66-kDa protein-encoding gene of Angiostrongylus cantonensis and Angiostrongylus malaysiensis
Source: Parasitology. 2022 Nov 2;150(1):98–114. doi: 10.1017/S0031182022001573 (PMC10090599; doi:10.1017/S0031182022001573)
Supplement: Supplementary file 1 [file S0031182022001573sup001.pdf]

Table S1. List of the specimens and their geographical origins.

Species	Code	Haplotype	Host	Country, region, province	GenBank accession number	Authors
A. cantonensis	AcF-1Cm	Ac66-1	*Bandicota indica*	Thailand, North, Chiang Mai	MH562054	Eamsobhana et al., 2019
	AcM-1Cm	Ac66-1	*Bandicota indica*	Thailand, North, Chiang Mai	MH562055	Eamsobhana et al., 2019
	AcM-24Rn	Ac66-1	*Rattus norvegicus*	Thailand, South, Ranong	MH562063	Eamsobhana et al., 2019
	AcM-1Rn	Ac66-1	*Rattus rattus*	Thailand, South, Ranong	MH562072	Eamsobhana et al., 2019
	AcF-2Sr	Ac66-1	*Bandicota indica*	Thailand, South, Surat Thani	MH562074	Eamsobhana et al., 2019
	AcF-13Bn	Ac66-1	*Rattus norvegicus*	Thailand, Central, Bangkok	MH562084	Eamsobhana et al., 2019
	AcM-14Lb	Ac66-1	*Rattus norvegicus*	Thailand, Central, Lopburi	MH562085	Eamsobhana et al., 2019
	AcM-2Psl	Ac66-1	*Rattus rattus*	Thailand, Central, Phitsanulok	MH562088	Eamsobhana et al., 2019
	AcF-3H	Ac66-1	*Rattus norvegicus*	Hawaii, lab strain	MH562089	Eamsobhana et al., 2019
	AcM-3H	Ac66-1	*Rattus norvegicus*	Hawaii, lab strain	MH562090	Eamsobhana et al., 2019
	AcF-3 T	Ac66-1	*Rattus norvegicus*	Thailand, lab strain	MH562091	Eamsobhana et al., 2019
	AcF-1 J	Ac66-1	*Rattus* sp.	Japan, Okinawa	MH562092	Eamsobhana et al., 2019
	AcM-2 J	Ac66-1	*Rattus* sp.	Japan, Okinawa	MH562093	Eamsobhana et al., 2019
	Ac42KPT_TH	Ac66-1	*Bandicota* sp.	Thailand, Central, Kamphaeng Phet	OM280392	present study
	Ac43KPT_TH	Ac66-1	*Bandicota* sp.	Thailand, Central, Kamphaeng Phet	OM280393	present study
	Ac47CPM_TH	Ac66-1	*Achatina fulica*	Thailand, Northeast, Chaiyaphum	OM280397	present study
	Ac49CPM_TH	Ac66-1	*Achatina fulica*	Thailand, Northeast, Chaiyaphum	OM280399	present study
	Ac50CPM_TH	Ac66-1	*Achatina fulica*	Thailand, Northeast, Chaiyaphum	OM280400	present study
	Ac51CPM_TH	Ac66-1	*Achatina fulica*	Thailand, Northeast, Chaiyaphum	OM280401	present study
	Ac52CPM_TH	Ac66-1	*Achatina fulica*	Thailand, Northeast, Chaiyaphum	OM280402	present study
	Ac53CPM_TH	Ac66-1	*Achatina fulica*	Thailand, Northeast, Chaiyaphum	OM280403	present study
	Ac54CPM_TH	Ac66-1	*Achatina fulica*	Thailand, Northeast, Chaiyaphum	OM280404	present study
	AcF-1Sr	Ac66-2	*Bandicota indica*	Thailand, South, Surat Thani	MH562073	Eamsobhana et al., 2019
	AcM-4Sp	Ac66-2	*Rattus rattus*	Thailand, Central, Samut Prakan	MH562081	Eamsobhana et al., 2019
	AcF-1Sp	Ac66-2	*Rattus rattus*	Thailand, Central, Samut Prakan	MH562082	Eamsobhana et al., 2019
	AcM-10Bn	Ac66-2	*Rattus norvegicus*	Thailand, Central, Bangkok	MH562083	Eamsobhana et al., 2019
	AcF-2 J	Ac66-2	*Rattus* sp.	Japan, Okinawa	MH562099	Eamsobhana et al., 2019
	Ac45CPM_TH	Ac66-2	*Achatina fulica*	Thailand, Northeast, Chaiyaphum	OM280395	present study

**Species**	**Code**	**Haplotype**	**Host**	**Country, region, province**	**GenBank accession number**	**Authors**
	Ac46CPM_TH	Ac66-2	*Achatina fulica*	Thailand, Northeast, Chaiyaphum	OM280396	present study
	Ac48CPM_TH	Ac66-2	*Achatina fulica*	Thailand, Northeast, Chaiyaphum	OM280398	present study
	Ac55CPM_TH	Ac66-2	*Achatina fulica*	Thailand, Northeast, Chaiyaphum	OM280405	present study
	AcF-2Psl	Ac66-3	*Rattus rattus*	Thailand, Central, Phitsanulok	MH562087	Eamsobhana et al., 2019
	AcF-10Sk	Ac66-4	*Rattus rattus*	Thailand, South, Song Khla	MH562068	Eamsobhana et al., 2019
	AcM-10Sk	Ac66-4	*Rattus rattus*	Thailand, South, Song Khla	MH562069	Eamsobhana et al., 2019
	AcM-1Kk	Ac66-4	*Rattus rattus*	Thailand, Northeast, Khon Kaen	MH562086	Eamsobhana et al., 2019
	AcF-2 T	Ac66-4	*Rattus norvegicus*	Thailand, lab strain	MH562102	Eamsobhana et al., 2019
	AcM-2 T	Ac66-4	*Rattus norvegicus*	Thailand, lab strain	MH562103	Eamsobhana et al., 2019
	AcM-1 J	Ac66-5	*Rattus* sp.	Japan, Okinawa	MH562097	Eamsobhana et al., 2019
	AcM-3 T	Ac66-6	*Rattus norvegicus*	Thailand, lab strain	MH562094	Eamsobhana et al., 2019
	AcF-1 T	Ac66-7	*Rattus norvegicus*	Thailand, lab strain	MH562095	Eamsobhana et al., 2019
	AcF-1H	Ac66-8	*Rattus norvegicus*	Hawaii, lab strain	MH562098	Eamsobhana et al., 2019
	AcM-1H	Ac66-9	*Rattus norvegicus*	Hawaii, lab strain	MH562096	Eamsobhana et al., 2019
	AcF-1C	Ac66-10	*Rattus* sp.	China, Guanhxi	MH562101	Eamsobhana et al., 2019
	AcF-3C	Ac66-10	*Rattus* sp.	China, Guanhxi	MH562104	Eamsobhana et al., 2019
	AcM-2C	Ac66-10	*Rattus* sp.	China, Guanhxi	MH562105	Eamsobhana et al., 2019
	AcM-2H	Ac66-10	*Rattus norvegicus*	Hawaii, lab strain	MH562108	Eamsobhana et al., 2019
	AcF-2H	Ac66-11	*Rattus norvegicus*	Hawaii, lab strain	MH562109	Eamsobhana et al., 2019
	AcF-6Pk	Ac66-12	*Rattus rattus*	Thailand, west, Prachuap Khiri Khan	MH562064	Eamsobhana et al., 2019
	AcM-6Pk	Ac66-12	*Rattus rattus*	Thailand, west, Prachuap Khiri Khan	MH562065	Eamsobhana et al., 2019
	AcF-2C	Ac66-12	*Rattus* sp.	China, Guanhxi	MH562100	Eamsobhana et al., 2019
	AcM-3C	Ac66-12	*Rattus* sp.	China, Guanhxi	MH562106	Eamsobhana et al., 2019
	AcM-1C	Ac66-13	*Rattus* sp.	China, Guanhxi	MH562107	Eamsobhana et al., 2019
	Ac44CPM_TH	Ac66-14	*Achatina fulica*	Thailand, Northeast, Chaiyaphum	OM280394	present study
A. malaysiensis	AmF1-5Bkk	Am66-1	*Rattus exulan*	Thailand, Central, Bangkok	MH562052	Eamsobhana et al., 2019

**Species**	**Code**	**Haplotype**	**Host**	**Country, region, province**	**GenBank accession number**	**Authors**
	AmF-1Cr	Am66-1	*Bandicota indica*	Thailand, North, Chiang Rai	MH562056	Eamsobhana et al., 2019
	AmM-1Cr	Am66-1	*Bandicota indica*	Thailand, North, Chiang Rai	MH562057	Eamsobhana et al., 2019
	AmM-2Kb	Am66-1	*Rattus norvegicus*	Thailand, West, Kanchanaburi	MH562062	Eamsobhana et al., 2019
	AmM-1 Tak	Am66-1	*Bandicota bengalensis*	Thailand, West, Tak	MH562077	Eamsobhana et al., 2019
	AmF-1Mh	Am66-1	*Rattus losea*	Thailand, North, Mae Hong Son	MH562080	Eamsobhana et al., 2019
	AmF-1Mal	Am66-1	*Rattus tiomanicus*	Malaysia, Pahang	MH562110	Eamsobhana et al., 2019
	AmM-1Mal	Am66-1	*Rattus tiomanicus*	Malaysia, Pahang	MH562111	Eamsobhana et al., 2019
	AmF-2Mal	Am66-1	*Rattus tiomanicus*	Malaysia, Pahang	MH562112	Eamsobhana et al., 2019
	AmM-2Mal	Am66-1	*Rattus tiomanicus*	Malaysia, Pahang	MH562113	Eamsobhana et al., 2019
	Am1PRE_TH	Am66-1	*Achatina fulica*	Thailand, North, Phrae	OM280406	present study
	Am2PRE_TH	Am66-1	*Achatina fulica*	Thailand, North, Phrae	OM280407	present study
	Am3PRE_TH	Am66-1	*Achatina fulica*	Thailand, North, Phrae	OM280408	present study
	Am14CRI_TH	Am66-1	*Achatina fulica*	Thailand, North, Chiang Rai	OM280419	present study
	Am15CRI_TH	Am66-1	*Achatina fulica*	Thailand, North, Chiang Rai	OM280420	present study
	Am18CRI_TH	Am66-1	*Achatina fulica*	Thailand, North, Chiang Rai	OM280423	present study
	Am25CRI_TH	Am66-1	*Achatina fulica*	Thailand, North, Chiang Rai	OM280430	present study
	Am28CRI_TH	Am66-1	*Achatina fulica*	Thailand, North, Chiang Rai	OM280433	present study
	Am29CRI_TH	Am66-1	*Achatina fulica*	Thailand, North, Chiang Rai	OM280434	present study
	Am34CRI_TH	Am66-1	*Achatina fulica*	Thailand, North, Chiang Rai	OM280439	present study
	AmF-10Nk	Am66-2	*Rattus norvegicus*	Thailand, Northeast, Nong Khai	MH562060	Eamsobhana et al., 2019
	AmM-10Nk	Am66-3	*Rattus norvegicus*	Thailand, Northeast, Nong Khai	MH562061	Eamsobhana et al., 2019
	AmF-1Nk	Am66-4	*Rattus norvegicus*	Thailand, Northeast, Nong Khai	MH562058	Eamsobhana et al., 2019
	AmM-38St	Am66-4	*Rattus rattus*	Thailand, South, Satun	MH562071	Eamsobhana et al., 2019
	AmF-1 Tak	Am66-4	*Bandicota bengalensis*	Thailand, West, Tak	MH562078	Eamsobhana et al., 2019
	AmM-2Mh	Am66-4	*Rattus losea*	Thailand, North, Mae Hong Son	MH562079	Eamsobhana et al., 2019
	AmM-1Png	Am66-5	*Rattus rattus*	Thailand, South, Phang Nga	MH562075	Eamsobhana et al., 2019
	AmM-2Png	Am66-5	*Rattus rattus*	Thailand, South, Phang Nga	MH562076	Eamsobhana et al., 2019
	AmF2-5Bkk	Am66-6	*Rattus exulans*	Thailand, Central, Bangkok	MH562053	Eamsobhana et al., 2019

Species	Code	Haplotype	Host	Country, region, province	GenBank accession number	Authors
	AmF-1 Tr	Am66-6	*Rattus rattus*	Thailand, South, Trang	MH562066	Eamsobhana et al., 2019
	AmF-11Tr	Am66-6	*Rattus rattus*	Thailand, South, Trang	MH562067	Eamsobhana et al., 2019
	AmF-38St	Am66-7	*Rattus rattus*	Thailand, South, Satun	MH562070	Eamsobhana et al., 2019
	AmM-1Nk	Am66-8	*Rattus norvegicus*	Thailand, Northeast, Nong Khai	MH562059	Eamsobhana et al., 2019
	Am4CRI_TH	Am66-9	*Achatina fulica*	Thailand, North, Chiang Rai	OM280409	present study
	Am5CRI_TH	Am66-9	*Achatina fulica*	Thailand, North, Chiang Rai	OM280410	present study
	Am6CRI_TH	Am66-9	*Achatina fulica*	Thailand, North, Chiang Rai	OM280411	present study
	Am7CRI_TH	Am66-9	*Achatina fulica*	Thailand, North, Chiang Rai	OM280412	present study
	Am9CRI_TH	Am66-9	*Achatina fulica*	Thailand, North, Chiang Rai	OM280414	present study
	Am11CRI_TH	Am66-9	*Achatina fulica*	Thailand, North, Chiang Rai	OM280416	present study
	Am12CRI_TH	Am66-9	*Achatina fulica*	Thailand, North, Chiang Rai	OM280417	present study
	Am13CRI_TH	Am66-9	*Achatina fulica*	Thailand, North, Chiang Rai	OM280418	present study
	Am19CRI_TH	Am66-9	*Achatina fulica*	Thailand, North, Chiang Rai	OM280424	present study
	Am22CRI_TH	Am66-9	*Achatina fulica*	Thailand, North, Chiang Rai	OM280427	present study
	Am23CRI_TH	Am66-9	*Achatina fulica*	Thailand, North, Chiang Rai	OM280428	present study
	Am24CRI_TH	Am66-9	*Achatina fulica*	Thailand, North, Chiang Rai	OM280429	present study
	Am26CRI_TH	Am66-9	*Achatina fulica*	Thailand, North, Chiang Rai	OM280431	present study
	Am27CRI_TH	Am66-9	*Achatina fulica*	Thailand, North, Chiang Rai	OM280432	present study
	Am30CRI_TH	Am66-9	*Achatina fulica*	Thailand, North, Chiang Rai	OM280435	present study
	Am31CRI_TH	Am66-9	*Achatina fulica*	Thailand, North, Chiang Rai	OM280436	present study
	Am33CRI_TH	Am66-9	*Achatina fulica*	Thailand, North, Chiang Rai	OM280438	present study
	Am35CRI_TH	Am66-9	*Achatina fulica*	Thailand, North, Chiang Rai	OM280440	present study
	Am39CRI_TH	Am66-9	*Achatina fulica*	Thailand, North, Chiang Rai	OM280444	present study
	Am8CRI_TH	Am66-10	*Achatina fulica*	Thailand, North, Chiang Rai	OM280413	present study
	Am20CRI_TH	Am66-10	*Achatina fulica*	Thailand, North, Chiang Rai	OM280425	present study
	Am21CRI_TH	Am66-10	*Achatina fulica*	Thailand, North, Chiang Rai	OM280426	present study
	Am32CRI_TH	Am66-10	*Achatina fulica*	Thailand, North, Chiang Rai	OM280437	present study
	Am36CRI_TH	Am66-10	*Achatina fulica*	Thailand, North, Chiang Rai	OM280441	present study

**Species**	**Code**	**Haplotype**	**Host**	**Country, region, province**	**GenBank accession number**	**Authors**
	Am37CRI_TH	Am66-10	*Achatina fulica*	Thailand, North, Chiang Rai	OM280442	present study
	Am38CRI_TH	Am66-10	*Achatina fulica*	Thailand, North, Chiang Rai	OM280443	present study
	Am40CRI_TH	Am66-10	*Achatina fulica*	Thailand, North, Chiang Rai	OM280445	present study
	Am41CRI_TH	Am66-10	*Achatina fulica*	Thailand, North, Chiang Rai	OM280446	present study
	Am10CRI_TH	Am66-11	*Achatina fulica*	Thailand, North, Chiang Rai	OM280415	present study
	Am16CRI_TH	Am66-12	*Achatina fulica*	Thailand, North, Chiang Rai	OM280421	present study
	Am17CRI_TH	Am66-13	*Achatina fulica*	Thailand, North, Chiang Rai	OM280422	present study
